# Clinical Predictors and Risk Factors of Gleason Score Upgrade: A Retrospective Cohort Analysis

**DOI:** 10.3390/diagnostics15101238

**Published:** 2025-05-14

**Authors:** Carmine Sciorio, Riccardo Giannella, Lorenzo Romano, Benito Fabio Mirto, Antonio Di Girolamo, Antonio Ruffo, Giuseppe Romeo, Fabio Esposito, Felice Crocetto, Luigi Napolitano, Raffaele Balsamo, Francesco Trama, Francesco Bottone, Carmelo Quattrone, Vittorio Imperatore, Lorenzo Spirito

**Affiliations:** 1 UOC Urologia, Ospedale Manzoni, 23900 Lecco, Italy; carmine.sciorio@gmail.com; 2 UOC Urologia, AORN “A. Cardarelli”, 80131 Napoli, Italy; riccardogiannella@libero.it (R.G.); gp.romeo@icloud.com (G.R.); 3 Department of Woman, Child and General and Specialized Surgery, Università degli Studi della Campania “Luigi Vanvitelli”, 81100 Napoli, Italy; lorenzo.romano@unicampania.it (L.R.); carmeloquattrone@hotmail.it (C.Q.); lorenzospirito@msn.com (L.S.); 4 Department of Neurosciences, Reproductive Sciences and Odontostomatology, University of Naples “Federico II”, 80131 Naples, Italy; felice.crocetto@unina.it; 5 UOC Urologia, AORN Moscati, 83100 Avellino, Italy; antonio.digirolamo@hotmail.it (A.D.G.); v.imperatore1@gmail.com (V.I.); 6 Dipartimento di Medicina e di Scienze della Salute “Vincenzo Tiberio”, UNIMOL, 86039 Termoli, Italy; antonio.ruffo7@gmail.com; 7 Urology Unit, Casa di Cura “Nostra Signora di Lourdes”, 80040 Napoli, Italy; info@fabioespositourologo.com; 8 Azienda Sanitaria Locale (ASL) Salerno, Via Vernieri, 84125 Salerno, Italy; nluigi89@libero.it; 9 UOC Urologia, Monaldi Hospital, 80131 Napoli, Italy; raffaelebalsamo5@gmail.com; 10 UOC Urologia, Asl Napoli 2 Nord, PO “Santa Maria delle Grazie”, 80078 Pozzuoli, Italy; francescotrama@gmail.com; 11 UOC Urologia, Università degli Studi della Campania “Luigi Vanvitelli”, 80138 Napoli, Italy; bottonefrancesco@yahoo.it

**Keywords:** prostate cancer, radical prostatectomy, Gleason score, Gleason score upgrade

## Abstract

**Background:** In prostate cancer (PCa) patients, discrepancies between biopsy-assigned Gleason Scores and those determined from surgical specimens are frequently reported. This phenomenon, known as Gleason score upgrade (GSU), can have significant clinical implications. This work aims to understand the factors contributing to GSU for refining prostate cancer management strategies. **Methods:** Data from 779 patients diagnosed with histologically confirmed PCa who underwent robot-assisted radical prostatectomy at a single tertiary care institution between January 2005 and December 2020 were examined. **Results:** In the univariable setting, 5-alpha reductase inhibitor (5-ARI) use was associated with a higher percentage of upgrading (42.3% vs. 30.4% among non-users; *p* = 0.03942). A more advanced pathological T stage (*p* = 0.01114) and lymph node positivity (*p* < 0.00001) correlated significantly with GSU. In the logistic regression model, advanced pathological stage increased the odds more than twofold (OR = 2.807, *p* = 0.00135). 5-ARI use was associated with notably higher odds of upgrading (OR = 3.809, *p* = 0.00004). Younger age slightly increased the likelihood of GSU (OR = 0.951 per year increase in age, *p* = 0.01101). **Conclusions:** Younger age, advanced pathological stage, and the use of 5-alpha reductase inhibitors were identified as significant predictors of GSU.

## 1. Introduction

Prostate cancer (PCa) is one of the most common malignancies affecting men globally, and it remains a leading cause of cancer-related morbidity and mortality, particularly in developed countries with aging populations. In Italy, prostate cancer represents a significant public health concern, with incidence rates closely mirroring trends observed in other Western countries [1,2]. As the population ages, the incidence of prostate cancer continues to rise, underscoring the need for effective diagnostic and therapeutic strategies. Radical prostatectomy (RP) is a primary curative treatment for localized prostate cancer, offering the potential for complete tumor eradication in patients with organ-confined disease. However, the success of this intervention is highly dependent on accurate preoperative assessment of tumor aggressiveness to guide treatment planning and prognostication [3,4,5].

A critical component in the evaluation of prostate cancer severity is the Gleason scoring system, which classifies tumors based on histological patterns observed under microscopy. The Gleason score (GS) remains the cornerstone of risk stratification, influencing both the choice of treatment and prognosis. However, discrepancies between biopsy-assigned Gleason scores and those determined from radical prostatectomy specimens are frequently reported. This phenomenon, known as Gleason score upgrade (GSU), occurs when the final pathology reveals a higher-grade tumor than was initially identified during the biopsy. GSU can have significant clinical implications, as it may necessitate more aggressive therapeutic approaches or the addition of adjuvant treatments, both of which can impact patient outcomes, including survival and quality of life [4,6].

Understanding the factors contributing to GSU is essential for refining prostate cancer management strategies. Various demographic and clinical variables have been implicated in influencing the likelihood of GSU. Age, for instance, has been studied extensively with mixed findings. Some research suggests that older patients are more likely to have less aggressive tumors identified on biopsy due to age-related tumor biology, while other studies propose that increased clinical vigilance in elderly patients could lead to more comprehensive sampling and accurate grading [7]. Additionally, serum prostate-specific antigen (PSA) levels, a widely used biomarker for prostate cancer detection and monitoring, and body mass index (BMI) have been explored as potential modifiers of GSU risk. Elevated PSA levels may prompt more aggressive diagnostic workups, potentially reducing the likelihood of undergrading [8,9,10]. Conversely, obesity has been linked to larger prostate volumes, which may hinder effective biopsy sampling and increase the chance of missing high-grade tumor foci [11,12].

Moreover, the widespread use of pharmacologic agents for benign prostatic hyperplasia (BPH) introduces additional complexity in preoperative tumor assessment. 5-alpha reductase inhibitors (5-ARIs), such as finasteride and dutasteride, inhibit the conversion of testosterone to dihydrotestosterone, leading to prostate volume reduction and lowering serum PSA levels by approximately 50%. While a smaller prostate may theoretically be easier to sample thoroughly, concerns have been raised that 5-ARIs might alter tumor histology, masking high-grade lesions and contributing to GSU [13]. The Prostate Cancer Prevention Trial (PCPT) highlighted this complexity, suggesting that while 5-ARIs reduce overall prostate cancer incidence, they might increase the detection of higher-grade tumors, sparking ongoing debate about their role in prostate cancer risk management.

Advancements in diagnostic imaging have provided clinicians with powerful tools to improve preoperative tumor characterization. Multiparametric magnetic resonance imaging (mpMRI) and prostate-specific membrane antigen positron emission tomography (PSMA PET) scans have revolutionized prostate cancer imaging, allowing for the more accurate detection of clinically significant disease and better assessment of local and distant tumor spread [14,15,16,17]. These modalities enhance risk stratification and can help to identify aggressive tumor features that traditional biopsy methods might miss. In Italy, adopting these advanced imaging techniques has contributed to more precise surgical planning and potentially mitigated the risk of GSU.

The surgical technique also plays a crucial role in managing prostate cancer, particularly concerning nerve-sparing (NS) approaches during radical robot-assisted radical prostatectomy. NS techniques aim to preserve erectile function and urinary continence by sparing the neurovascular bundles surrounding the prostate [18,19,20,21]. However, there is an ongoing debate about whether NS procedures compromise oncological outcomes by increasing the risk of positive surgical margins, especially in patients with high-risk diseases [22,23,24]. Some studies suggest that NS surgery, when performed in appropriately selected patients, does not necessarily increase the likelihood of leaving behind residual cancer or contribute to GSU [25]. Nonetheless, in cases of aggressive pathological features such as seminal vesicle invasion or extracapsular extension, the balance between functional preservation and oncological control becomes particularly delicate [26].

The current study seeks to deepen our understanding of GSU by retrospectively analyzing data from a larger cohort of patients undergoing robot-assisted radical prostatectomy. The primary objective of this analysis is to determine whether clinical variables—specifically age, preoperative PSA levels, and 5-ARI therapy—are significantly correlated with the likelihood of GSU. By controlling for pathological stage and baseline clinical risk categories, the study aims to clarify whether these factors independently contribute to Gleason score discrepancies [27].

Ultimately, this research seeks to inform clinical practice by identifying modifiable risk factors for GSU and improving preoperative risk stratification. Understanding how medications, patient demographics, and surgical techniques interact to affect tumor grading can guide more tailored diagnostic and therapeutic strategies, reducing the occurrence of unexpected high-grade tumors at final pathology [28].

## 2. Patients and Methods

### 2.1. Overall Study Design

This retrospective cohort study examined clinical data from patients diagnosed with histologically confirmed prostate cancer who underwent robot-assisted radical prostatectomy (RARP) at a single tertiary care institution between January 2005 and December 2020. The study specifically included patients with confirmed prostate adenocarcinoma who received RARP as their primary treatment, while those with incomplete clinical or pathological data were excluded to ensure data integrity.

The primary objective of this research was to identify factors that may predict Gleason score upgrading (GSU) following RARP. To achieve this, this study employed both univariate and multivariable logistic regression analyses to explore relationships between various clinical and pathological characteristics and the likelihood of Gleason score changes. All patient records underwent thorough review and data cleaning to maintain accuracy, with any incomplete cases being systematically excluded.

Gleason score upgrading was defined by comparing biopsy results to post-surgical pathology. Patients whose Gleason score increased after surgery were classified in the “Upgrade” group, whereas those whose scores remained unchanged were placed in the “No Upgrade” group. The analysis included a range of clinical and pathological factors. Continuous variables such as preoperative prostate-specific antigen levels, age at diagnosis, tumor length, and prostate weight were assessed alongside categorical variables like pathological tumor stage, the use of alpha-reductase inhibitors, and lymph node involvement.

This study was conducted in accordance with the Declaration of Helsinki on ethical principles for medical research involving human subjects, and each patient provided written informed consent to participate. Ethics committee approval was obtained prior to patient enrollment.

### 2.2. Statistical Analysis

Descriptive statistics were calculated to summarize patient demographics and clinical variables, with continuous variables expressed as medians and interquartile ranges (IQR) and categorical variables reported as frequencies and percentages. The Mann–Whitney U test was used to compare medians between the ‘Upgrade’ and ‘No Upgrade’ groups for continuous variables due to their non-normal distribution. For categorical variables, Fisher’s Exact Test was applied to 2 × 2 contingency tables, particularly for the lymph node positivity variable, given the small sample sizes, while the Chi-Square Test of Independence was used for categorical variables with more than two categories, such as pathological tumor stage. Univariate analyses were conducted to assess associations between variables and Gleason score upgrading, and a multivariable logistic regression analysis was performed to identify independent predictors. Statistical significance was determined using a two-sided alpha level of 0.05, with *p*-values less than 0.05 considered statistically significant.

## 3. Results

### 3.1. Study Population

**Figure 1:**
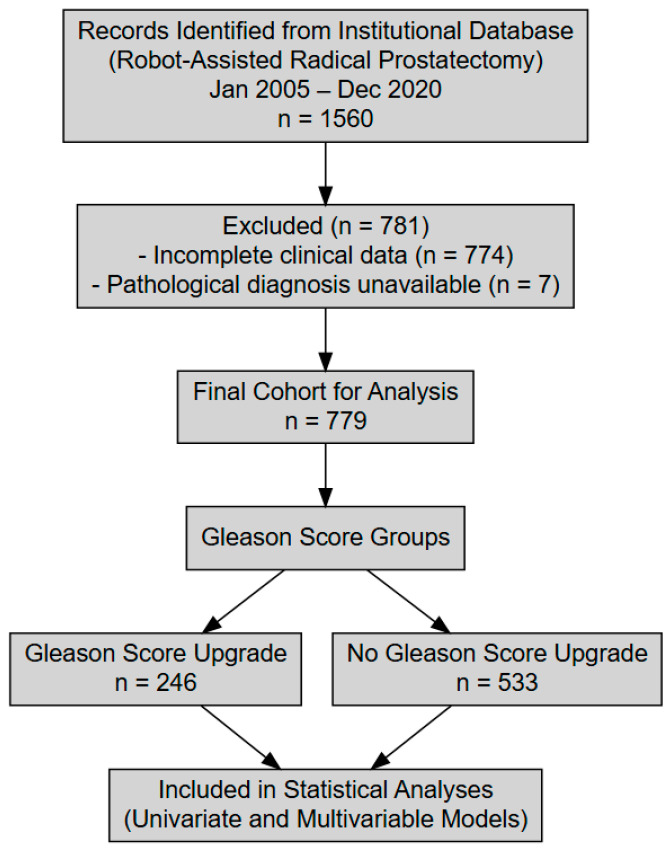
CONSORT-style flow diagram illustrating patient selection and inclusion criteria.

A total of 779 patients formed the study cohort, with each undergoing robot-assisted radical prostatectomy for histologically confirmed prostate cancer between January 2005 and December 2020 (Figure 1). The mean (±SD) age at surgery was 63.46 ± 6.48 years (range: 43–85), and their mean preoperative PSA was 11.85 ± 7.63 ng/mL (range: 2.00–34.00). Tumor length information was available for 706 patients, yielding an average (±SD) of 17.47 ± 21.43 mm, while prostate weight was recorded for 300 patients at a mean (±SD) of 42.17 ± 22.98 g. Nerve-sparing procedures were performed in 55% of the total cohort (Table 1). Comparing biopsy-based Gleason scores with final pathology, 246 patients (31.58%) exhibited a Gleason score upgrade (GSU), while 533 (68.42%) maintained the same Gleason category (Table 2). The interval between prostate biopsy and robot-assisted radical prostatectomy was less than 6 months in all patients included in this study, ensuring a consistent and clinically relevant timeframe for assessing Gleason score upgrading. Among the patients analyzed, Gleason score upgrades occurred with varying frequencies. The most frequent change was from Gleason 6 to 7, observed in 58.37% of patients. Other notable upgrades included 8 → 9 in 14.29%, 7 → 8 in 8.16%, and 7 → 9 also in 8.16% of cases. The upgrade from 6 to 8 was seen in 7.76% of patients, while 6 → 9 occurred in 2.86%. A small proportion of patients (0.41%) experienced an upgrade from 6 to 10.

### 3.2. Univariable Predictors of Gleason Upgrade

In the univariable setting, significant differences emerged for several factors. Patients with a Gleason upgrade had a median age of 63 years, compared to 64 years in those without upgrading (*p* = 0.00394). Tumor length also differed, with an approximate median of 7 mm in the Upgrade group versus 12 mm in the No Upgrade group (*p* = 0.00002). Although the mean preoperative PSA was around 10 ng/mL in both groups, the difference did not reach statistical significance (*p* = 0.12279) (Table 3). In terms of categorical variables, 5-alpha reductase inhibitor use was associated with a higher percentage of upgrading (42.3% vs. 30.4% among non-users; *p* = 0.03942). A more advanced pathological T stage (e.g., pT3) correlated significantly with GSU (*p* = 0.01114), and lymph node positivity likewise demonstrated a notable association (*p* < 0.00001) (Table 4).

### 3.3. Multivariable Analysis

A logistic regression model identified three independent predictors of Gleason score upgrade after recoding the pathological T stage into pT2 versus pT3/pT4. First, an advanced pathological stage increased the odds more than twofold (OR = 2.807, *p* = 0.00135). Second, 5-alpha reductase inhibitor use was associated with notably higher odds of upgrading (OR = 3.809, *p* = 0.00004), suggesting that patients on these medications may require especially careful biopsy evaluation. Third, younger age slightly increased the likelihood of GSU (OR = 0.951 per year increase in age, *p* = 0.01101), indicating that older patients were relatively less likely to undergo Gleason upgrade. Tumor length (OR = 0.989, *p* = 0.10731) and lymph node positivity (OR = 0.950, *p* = 0.52735) were not significant predictors in the multivariable setting once other variables were accounted for (Table 5).

## 4. Discussion

The results of this study provide valuable insights into the multifaceted dynamics influencing Gleason score upgrade (GSU) in prostate cancer (PCa) patients undergoing robot-assisted radical prostatectomy (RARP). Notably, this study identifies younger age, advanced pathological stage, and the use of 5-alpha reductase inhibitors (5-ARIs) as significant predictors of GSU. These findings contribute to the existing body of literature by offering a deeper understanding of how these factors individually and collectively impact tumor grading and clinical decision-making. By integrating these findings with recent studies from PubMed, we can further explore potential mechanisms underlying GSU and discuss future directions for clinical management and research.

One noteworthy finding of this study is the observed association between younger patient age and the likelihood of GSU. This aligns with prior research suggesting that younger men may present with more biologically aggressive prostate tumors that are not fully captured by initial biopsy assessments [7]. Younger patients often harbor high-grade, poorly differentiated tumors, which might be under-sampled during routine biopsies. Furthermore, younger patients may undergo more conservative biopsy regimens due to their perceived lower risk, potentially leading to the underestimation of tumor grade. This observation suggests that more rigorous preoperative assessments for younger patients could be beneficial, including advanced imaging techniques such as multiparametric magnetic resonance imaging (mpMRI) and targeted biopsy strategies to reduce the risk of GSU [29].

The impact of 5-ARIs, such as finasteride and dutasteride, on GSU presents a complex and somewhat controversial picture. The multivariable analysis in this study identified 5-ARI use as a significant predictor of GSU, suggesting that patients on these medications might experience an underestimation of tumor grade. 5-ARIs function by inhibiting the conversion of testosterone to dihydrotestosterone (DHT), resulting in prostate volume reduction and decreased serum prostate-specific antigen (PSA) levels by approximately 50% [13]. While this volume reduction should theoretically improve biopsy sampling efficiency, concerns have been raised that 5-ARIs might alter tumor architecture, potentially masking high-grade lesions.

Several studies corroborate this finding. Eastham et al. [28] demonstrated that men diagnosed with prostate cancer while on finasteride therapy exhibited significant GSU at final pathology, underscoring the potential for underestimation during biopsy. Similarly, the Prostate Cancer Prevention Trial (PCPT) indicated that although 5-ARIs reduced overall prostate cancer incidence, they paradoxically increased the detection of high-grade tumors, possibly due to improved biopsy sampling in smaller prostates [13]. However, Ford et al. [30] questioned whether this apparent increase in high-grade tumors represented true biological progression or a diagnostic artifact resulting from prostate shrinkage.

Further complicating this relationship, Kusic et al. [31] reported that continuous finasteride therapy could induce neuroendocrine differentiation in prostate tissue, potentially contributing to a more aggressive tumor phenotype. This raises concerns that 5-ARIs might not merely obscure high-grade disease but could also influence tumor biology in ways that promote aggressiveness. Additionally, Hou et al. [32] observed that short-term dutasteride intake in patients with early-stage prostate cancer influenced biopsy outcomes, suggesting that even brief exposure to 5-ARIs might affect tumor detection and grading.

Another relevant study by Stephenson et al. [33] found that 5-ARI use impacted disease reclassification in men on active surveillance for localized prostate cancer. Specifically, patients on 5-ARIs had a higher likelihood of being reclassified to higher-risk categories, reinforcing the need for cautious monitoring of these patients. These findings collectively suggest that while 5-ARIs offer therapeutic benefits for benign prostatic hyperplasia (BPH), their effects on prostate cancer detection and grading warrant careful consideration.

The advanced pathological stage (pT3/pT4) was also identified as an independent predictor of GSU in this study [34]. Tumors exhibiting extracapsular extension or seminal vesicle invasion are inherently more aggressive and often present with heterogeneous histological patterns, increasing the risk of biopsy under-sampling [35]. This finding underscores the potential value of integrating advanced imaging techniques into preoperative evaluations. The use of mpMRI and prostate-specific membrane antigen positron emission tomography (PSMA PET) has been shown to improve the detection of clinically significant disease and better delineate tumor extent [14,15,16,17]. Incorporating these modalities could enhance risk stratification and help to identify patients at increased risk of GSU, enabling more tailored biopsy strategies and surgical planning.

Interestingly, this study found an inverse association between tumor length and GSU, suggesting that smaller or multifocal tumors may be more challenging to sample accurately during biopsy. This observation underscores the need for optimized biopsy protocols, particularly in detecting small-volume or multifocal disease. Targeted fusion biopsies, guided by mpMRI, have shown promise in improving the detection of clinically significant prostate cancer and could be instrumental in mitigating GSU in these cases [36,37,38].

Contrary to some concerns, this study did not find a significant independent effect of nerve-sparing (NS) surgical techniques on GSU. This finding aligns with previous research indicating that NS robot-assisted radical prostatectomy, when carefully performed in appropriately selected patients, does not compromise oncological outcomes [25,26,39]. However, in high-risk patients, the decision to preserve the neurovascular bundles must be made judiciously to balance functional preservation with complete cancer excision.

These findings may have important clinical implications. To mitigate the risk of underestimating tumor grade, patients receiving 5-ARI therapy should undergo more detailed preoperative assessments. This might involve integrating advanced imaging techniques, adopting more aggressive biopsy protocols, and closely monitoring PSA kinetics. Additionally, incorporating molecular profiling and emerging biomarkers into preoperative evaluations could refine risk stratification models and inform more personalized treatment strategies [40].

Future research should focus on large-scale, prospective studies to validate these findings and explore additional variables influencing GSU. For instance, investigating the impact of genetic mutations, tumor microenvironment interactions, and systemic factors such as metabolic syndrome or the prostate–gut microbiome could yield valuable insights into the mechanisms driving GSU [41]. Moreover, developing predictive models that integrate clinical, imaging, and molecular data could significantly improve the accuracy of GSU prediction and facilitate more effective clinical decision-making [42,43].

However, this study is not without limitations. Its retrospective design and single-institution setting may limit the generalizability of the findings. The observational nature of the data also precludes definitive causal inferences regarding the effects of 5-ARI use on GSU. Additionally, unmeasured confounding factors, such as variations in biopsy techniques and surgeon expertise, could influence the results. Despite these limitations, this study offers valuable insights into the predictors of GSU and underscores the need for comprehensive risk assessment strategies in prostate cancer management.

## 5. Conclusions

In conclusion, this study highlights the multifactorial nature of the Gleason score upgrade in prostate cancer patients undergoing robot-assisted radical prostatectomy. Younger age, advanced pathological stage, and the use of 5-alpha reductase inhibitors are identified as significant predictors of GSU. These findings emphasize the importance of individualized patient assessment, incorporating advanced imaging, optimized biopsy protocols, and careful consideration of medication use to improve preoperative risk stratification. Future prospective studies and multicenter collaborations are essential to validate these findings and develop innovative strategies to mitigate the risk of GSU, ultimately enhancing personalized care for men with prostate cancer.

## Figures and Tables

**Table 1:** Data availability per variable.

Variable	Available Data Count
Preoperative PSA	779
Age	779
Tumor length	706
Prostate weight	300
pT (pathological T stage)	779
Alpha (5-alpha reductase inhibitor use or not)	779
Lymph node positivity	411

**Table 2:** Gleason upgrade status.

Gleason Upgrade Status	Count	Percentage (%)
No Upgrade	533	68.42
Upgrade	246	31.58

**Table 3:** Median values of continuous variables stratified by GSU status.

Variable	No Upgrade Median (*n*)	Upgrade Median (*n*)	*p*-Value
Preoperative PSA	10.00 (533)	9.35 (246)	0.12279
Age	64.00 (533)	63.00 (246)	0.00364 *
Tumor length	12.00 (500)	7.00 (206)	0.00002 *
Prostate weight	38.00 (246)	33.50 (54)	0.11716

* Statistically significant at *p* < 0.05.

**Table 4:** Categorical variables by GSU status.

Variable	Category	No Upgrade *n* (%)	Upgrade *n* (%)	*p*-Value
Pathological T stage (pT)	pT2	178 (77.0)	53 (23.0)	0.01114 *
	pT3a	253 (64.4)	140 (35.6)	
	pT3b	96 (64.9)	52 (35.1)	
	pT4	6 (85.7)	1 (14.3)	
5-alpha reductase inhibitor use	No (0.0)	488 (69.6)	213 (30.4)	0.03932 *
	Yes (1.0)	45 (57.7)	33 (42.3)	
Lymph node positivity	No	289 (81.6)	65 (18.4)	<0.01 *
	Yes	244 (57.4)	181 (42.6)	

* Statistically significant at *p* < 0.05.

**Table 5:** Logistic regression analysis for Gleason upgrade.

Variable	Odds Ratio (OR)	95% CI	*p*-Value
Intercept	2.626	—	0.45248
(pT3/pT4 vs. pT2)	2.807	—	0.00135 *
Use of 5-alpha reductase inhibitors (Yes vs. No)	3.809	—	0.00004 *
Age	0.951	—	0.01101 *
Tumor length	0.989	—	0.10731
Lymph node positivity	0.950	—	0.52735

* Statistically significant at *p* < 0.05.

## Data Availability

New data were created, while data from records are unavailable due to privacy or ethical restrictions for maintaining the complete anonymity.
